# GlobalBuildingMap — Unveiling the mystery of global buildings

**DOI:** 10.1038/s41597-026-06578-9

**Published:** 2026-01-16

**Authors:** Xiao Xiang Zhu, Qingyu Li, Yilei Shi, Yuanyuan Wang, Adam J. Stewart, Jonathan Prexl, Fahong Zhang

**Affiliations:** 1 Chair of Data Science in Earth Observation, Technical University of Munich, Munich, Germany; 2 Munich Center for Machine Learning, Munich, Germany; 3 School of Engineering and Design, Technical University of Munich, Munich, Germany

**Keywords:** Urban ecology, Geography

## Abstract

Understanding how buildings are distributed globally is crucial to revealing the human footprint on our home planet. This built environment affects local climate, land surface albedo, resource distribution, and many other key factors that influence well-being and human health. Despite this, quantitative and comprehensive data on the distribution and properties of buildings worldwide is lacking. Using a big data analytics approach and nearly 800,000 satellite images, we generated the highest resolution and highest accuracy building map ever created: the GlobalBuildingMap (GBM). A joint analysis of building maps and solar potentials indicates that rooftop solar energy can supply the global energy consumption need at a reasonable cost. Specifically, if solar panels were placed on the roofs of all buildings, they could supply 1.1–3.3 times — depending on the efficiency of the solar device — the global energy consumption in 2020.

## Background & Summary

Identifying all buildings on the planet would further a better understanding of human activities across the globe, which is crucial for making key strategic decisions needed to tackle the grand societal challenges of the century, such as urbanization and climate change, and further analyze their impacts. For example, more than half the population of the planet (currently about 56%) live in urban areas^1^. This built environment influences local and regional weather and climate, water runoff characteristics and flood risk, energy efficiency, resource allocation and distribution, and numerous other factors that affect well-being. The characteristics of the built environment such as building density, green spaces, traffic flow, and so on have a profound impact, therefore, on some four billion people. Robust and comprehensive information on these characteristics and how they change with time is essential for urban planning and modeling impacts.

In the context of climate change, the latest 2022 IPCC assessment report (AR6)^2^ shows that global net anthropogenic greenhouse gas (GHG) emissions in 2019 reached about 60 GtCO_2_-eq, 12% higher than that in 2010, which is the highest increase in average decadal emissions on record. Buildings contributed 21% of the GHG emissions in 2019. The expansion of human settlements naturally increases GHG emissions, due to the increase of population and production activities, as well as the loss of habitat, biomass, and carbon storage^3^. On the other hand, compact urban structures can also positively contribute to reducing GHG emissions^4^. IPCC AR6 included a separate chapter on buildings and suggested that new buildings and existing ones, if retrofitted, are projected to approach net zero GHG emissions in 2050 if ambitious renewable energy measures are implemented^2^. Therefore, applying a globally consistent mapping approach to understand the human settlement expansion can significantly contribute to the 2050 net zero carbon goal.

Given the urgent need, a number of building datasets have been created, as documented in Table 1. To date, the only complete global datasets include the Global Urban Footprint (GUF)^5^, High-Resolution Settlement Layer (HRSL)^6^, Global Human Settlement Layer (GHSL)^7^, and World Settlement Footprint (WSF)^8^. However, all of these raster datasets lack the spatial resolution necessary to identify smaller buildings or to generate vector data for individual buildings. Even though buildings only occupy a relatively small fraction of the land surface, they drive global environmental changes. Understanding the expansion pattern both broadly and down to the detail of individual buildings enables unprecedented applications. For example, the literature shows that compact urban structures contribute to reducing greenhouse gas emissions^4^, but could also worsen the urban environment through the urban heat island effect. A global building map also lays the foundation for creating global 3D building models for better study of the above-mentioned points, as multiple papers demonstrate the importance of including a vertical dimension in the building map^9–11^. We believe the location and extent of individual buildings are crucial to revealing the true footprint of humankind.

**Table 1:** Comparison between different building products.

Product	Spatial coverage	Completeness	Temporal coverage	Spatial resolution	Format
GUF^5^	Global	Complete	2011	12 m	Raster
HRSL^6^	Global	Complete	2015	30 m	Raster
GHSL^7^	Global	Complete	2018	10 m	Raster
WSF^8^	Global	Complete	2019	10 m	Raster
WSF 3D^10^	Global	Complete	2011–2013	90 m	Raster
OSM^12^	Global in part	Incomplete	Unknown	Unknown	Vector
Google^15,16^	Global South	Complete	Unknown	0.5 m	Vector
Microsoft^14^	Global in part	Incomplete	2014–2023	0.3–0.6 m	Vector
3D-GloBFP^11^	Global in part	Complete	2019–2021	Unknown	Vector
GBM	Global	Complete	2018–2019	3 m	Raster

GBM has the highest resolution and highest accuracy of any complete global building map.

Current efforts toward mapping individual buildings on a large scale are led, for example, by OpenStreetMap (OSM), Microsoft, and Google. However, there is a large gap between tailored local-/regional-scale mapping and systematic global-scale mapping of buildings. Both the completeness and coverage of the existing building maps are limited. OSM^12^ is the largest open database of building maps, but mainly covers Europe and part of North America. The coverage in Asia, Africa, South America, and Oceania is opportunistic since OSM includes only voluntarily contributed geographic information. This also leads to inconsistent quality across different countries and regions^13^. The Microsoft Building Footprint^14^ dataset covers most of the globe except for China, but is largely incomplete. Google’s Open Buildings^15^ and Open Buildings 2.5D^16^ datasets cover Africa, South Asia, Southeast Asia, Latin America, and the Caribbean (collectively referred to as the Global South). As of March 2025, OSM contains 600M buildings, Microsoft has mapped 1.4B buildings, and Google has mapped 1.8B buildings. None of the above-mentioned endeavors alone or their combinations offers global-scale building maps.

### GlobalBuildingMap

The availability of high-resolution, near global coverage satellite imagery, as well as recent technological advancements in data science, present the opportunity to close this knowledge gap. Here, we present the GlobalBuildingMap (GBM): the highest accuracy and highest resolution global building map ever created. GBM is derived from nearly 800,000 PlanetScope satellite images, and is distributed in the form of a binary raster (building and non-building) at a resolution of 3 meters. Such high spatial resolution is crucial for detecting smaller buildings and temporary shelters.

The automatic building segmentation framework shown in Fig. 5 is based on deep learning techniques. We collected over 100,000 pairs of OSM building footprint masks and PlanetScope satellite data, each with a size of 256  × 256 pixels, selected from 74 cities across the globe, paying special attention to areas with scarce geoinformation, such as Africa and Latin America. Among them, 20% were selected as validation samples to evaluate the performance of the trained models. We trained four state-of-the-art deep learning models^17–19^ with the prepared training dataset and fused their results by majority voting: a building pixel is determined if at least two of the four models predict it to be a building pixel. The trained models were employed for the inference of the global PlanetScope data by means of a complete pipeline of data download, analysis-ready data preparation, inferencing, and post-processing. We used the Global Urban Footprint (GUF)^5^ to detect settlement areas on a 0.2°  × 0.2° grid. However, we posit that our building map does not depend on the quality of the GUF, because even if only a single pixel of a grid cell appears as built in GUF, we will process the entire grid cell with our pipeline. Planet APIs were employed to acquire either Surface Reflectance or Basemap images, depending on the cloud coverage. Those images were calibrated and mosaicked for each grid cell. The inferencing step then took those mosaics and four pretrained models as input, and produced the raw global building footprints. As the last step, the raw global building footprints were filtered by removing false alarms using land cover layers, including WSF^20^ and FROM-GLC10^21^.

In total, the global building areas shown in the GBM amount to 0.67 million km^2^, 2.35 times more than the 0.2 million km^2^ estimated by Joshi *et al*.^22^. The latter estimate was made by means of a machine learning-based regression model trained using regional reference data collected from different data sources. This means, different from our approach which segments out individual buildings with a spatial resolution of 3 m and then sums building area, building areas are directly estimated at a resolution of 10 km. Besides the limited accuracy and generalizability of regression models, the underestimation of building areas from Joshi *et al*.^22^ could also be associated with a bias in the training data: buildings that were constructed after the year 2015 were discarded in the training data, and the reference data are not representative enough, as regions with sparse rooftops were not considered during model training. This confirms the importance of the more precise approach used in this study, in which individual buildings are detected, to avoid possible bias.

**Fig. 1:**
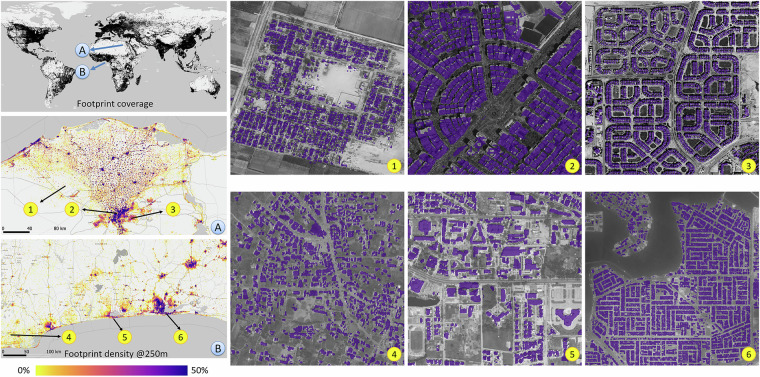
A glance at the GlobalBuildingMap. The black area in the upper left subfigure shows the coverage of the GBM. Two areas, (**A**) Cairo, Egypt and (**B**) Lagos, Nigeria, are exemplified by showing their building footprint density as a percentage in a 250 m  × 250 m patch, and their detailed individual building footprints at the city block level. It demonstrates that GBM not only shows the clustering of human settlements, but also clearly indicates individual buildings. Background images © Bing Maps.

Figure 1 shows the coverage of the GBM and a few examples of the building density and building footprint in Africa, where detailed building information is scarce. Locations (A) and (B) illustrate the building density of areas around Cairo, Egypt and Lagos, Nigeria, derived from the GBM by counting the percentage of 3 m  × 3 m building pixels within every 250 m  × 250 m patch. Different urban morphologies can be observed for those two urban agglomerations, as seen in locations (1–6). It also demonstrates that the GBM can provide detailed information at the individual building level.

**Fig. 2:**
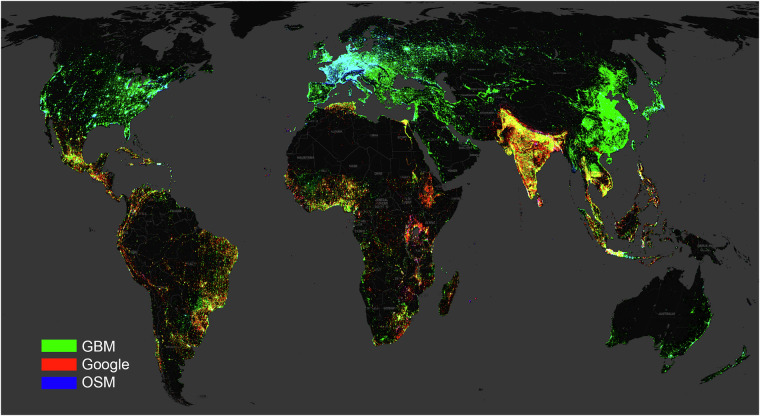
Global building density from three sources: our GBM (green), Google (red), and OSM (blue). Pure RGB color indicates only one source is available. Blended colors, including cyan, yellow, magenta, and white, indicate multiple sources are available. It can be seen that our footprint is the only source covering the entire globe. Google is only available in southern regions, and has a slight advantage in a few spots in eastern Africa and India, as red dominates those regions. OSM aligns well with our building footprint in Europe, as well as some Asian and North American cities, but is not available elsewhere. Only GBM covers some of the most populous regions in the world in East Asia. Background map © CartoDB.

Figure 2 draws a clear picture of the coverage of the building footprint provided by Google (red), GBM (green), and OSM (blue). A pure red, green, or blue color indicates that only one building data source out of the three is available, namely Google, GBM (ours), or OSM, respectively. Blended colors, including cyan, yellow, magenta, and white indicate that multiple data sources are available. In particular, cyan means that both OSM and GBM are available, yellow means that both Google and GBM are available, magenta shows that only Google and OSM are available, while white indicates that all data sources are available. It is apparent that GBM is the only source that has global coverage. Google is only available in southern regions and has a certain advantage in some spots (red in color) of eastern Africa and India. OSM matches well with our GBM in Europe, as well as some Asian and North American cities, but it is generally not available elsewhere. Among these data sources, the most populous regions in the world in East Asia are only covered by the GBM.

**Fig. 3:**
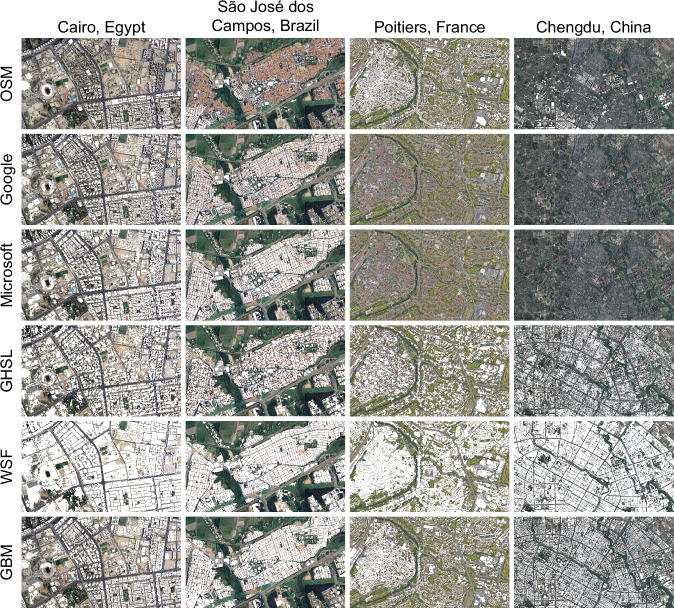
Among various data sources, our GBM shows globally consistent building maps on a building instance level. From the top to bottom are results (in white) obtained by OSM, Google, Microsoft, GHSL, WSF, and GBM. Background images © Google Maps.

For a closer look at the accuracy of GBM compared to other building products, Fig. 3 provides a direct comparison for cities from four different continents. OSM has close to complete coverage of Poitiers, France, but only partial coverage of all other cities. Google and Microsoft have good coverage over Cairo, Egypt and São José dos Campos, Brazil, but little to no coverage in other regions. Although GHSL and WSF have complete global coverage, they tend to overestimate building area. Only GBM is able to capture the fine details of smaller buildings interspersed by roads and vegetation.

The resulting high quality global building map unveils for the first time the mystery of global buildings, including their global distribution and density. It offers valuable new capabilities in downstream analysis, such as urban structure, solar potential of building areas, vulnerability analysis, population density aggregation, risk assessment, and urban planning.

### Solar Potential Analysis

The European Green Deal^23^ was announced by the European Commission in 2019, and is charged with addressing climate change and promoting sustainable development, with the goal of making the European Union climate neutral by 2050. To this end, fossil fuel and nuclear power plants need to be completely replaced by clean energy. In particular, the recent sanctions related to the conflict between Russia and Ukraine have led to a further energy crisis, confirming the importance of developing new and sustainable energy sources. Also, a recent study by Luderer *et al*.^24^ reported that renewables-based electrification plays a greater role in decarbonization than expected.

Solar energy is clean and renewable. IPCC AR6 shows that solar energy leads all mitigation strategies studied in the report in the potential and cost efficiency of reducing net emissions (by 2030) by a significant margin^2^. Estimates show that on average, 4.5 GtCO_2_-eq per year can be achieved with manageable cost. If every house in the world had solar panels on its roof, would the solar energy generated be sufficient to meet the global energy demand? The question can be easily answered by a joint analysis of the building footprint in GBM and solar potential provided by the Global Solar Atlas^25^. The annual solar potential *P* in a given location (*x*, *y*) can be formulated as:

1 $${P}_{x,y}={{\rm{PV}}}_{x,y}\ast (1-{\rm{loss}})\ast {N}_{p}\ast {N}_{d},$$

where PV_*x*,*y*_ is the photovoltaic (PV) power potential of a 1 kW-peak PV system at this location, which is a long-term yearly average of daily totals. *N*_*d*_ is the number of days in a year, i.e., 365. *N*_*p*_ is the effective number of 1 kW-peak PV systems, which is defined as *N*_*p*_ = *A*_*b*_/*A*_*p*_. The term *A*_*b*_ is the total area of global building roofs, whereas *A*_*p*_ is the area occupied by a 1 kW-peak PV system, which is set in the range of 10–30 m^2^, depending on the specifications of the solar device. Here we have chosen a range with a reasonable price–performance ratio that would be suitable for large-scale installation. In addition, losses due to dirt and soiling, mismatch, transformer, DC and AC cabling losses, and downtime are considered in the calculation. The average loss for different installation types is set to be 10%. For more details on our calculations, we recommend interested readers consult Solargis^25^.

**Fig. 4:**
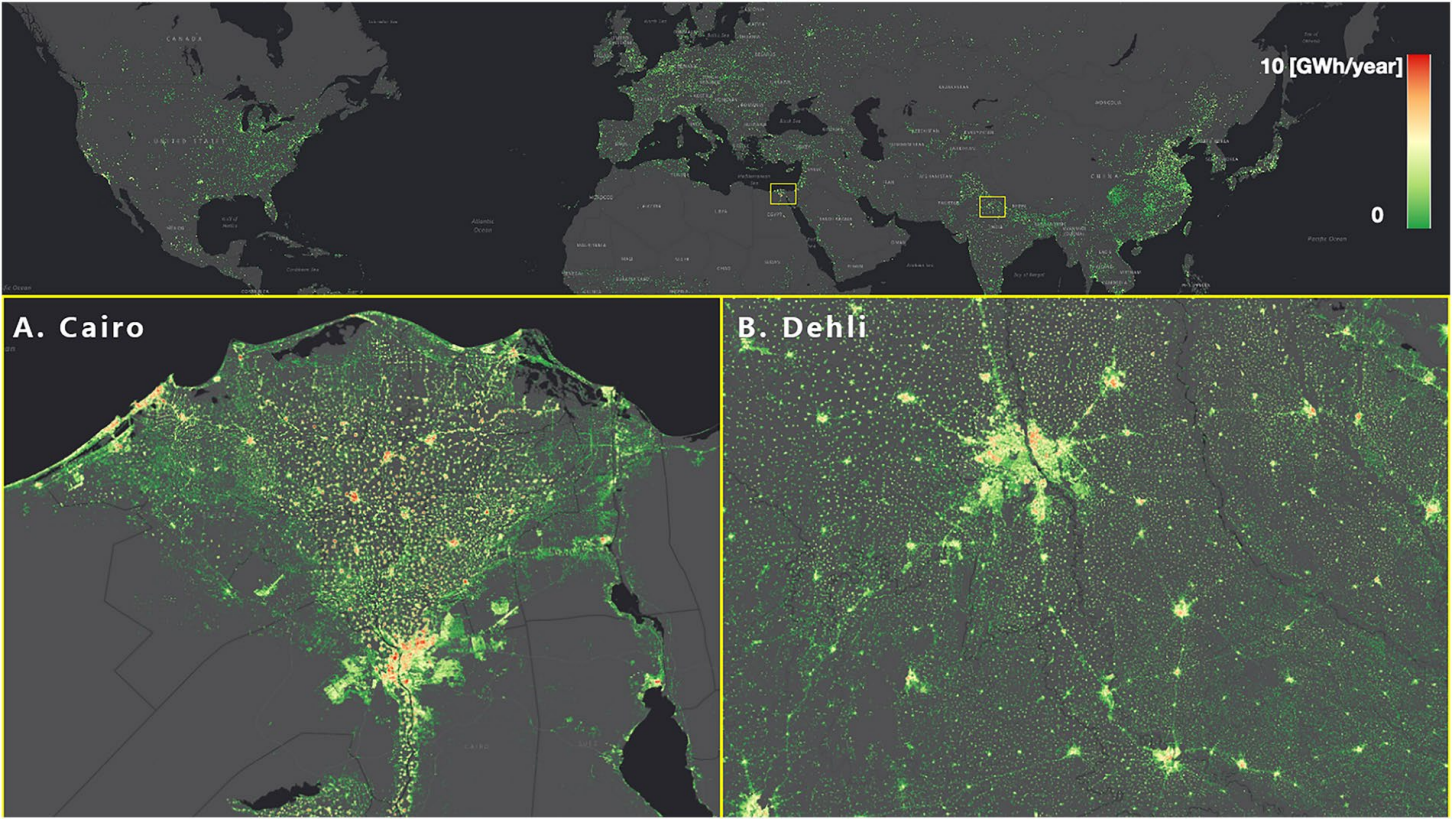
Rooftop solar potential analysis of global buildings. Color indicates the yearly solar potential per pixel in the range of 0–10 GWh/year with a spatial resolution of 250 m × 250 m. Two cities that are ideal for placing rooftop solar panels, (**A**). Cairo, Egypt and (**B**). Delhi, India, are zoomed in.

Accordingly, we estimate the yearly global rooftop solar potential to be 28–84 PWh, which is 1.1–3.3 times the global energy consumption of 2020 — the year with the highest energy consumption in human history. Given that the efficiency of the solar panel is assumed to be moderate in this estimate, it would be safe to state that if solar panels were placed on all building roofs, it would be possible to supply the global energy consumption need at a reasonable cost. This offers a promising perspective on promoting rooftop solar PVs across the globe, which currently account for 40% of the installed capacity of global solar PVs and 25% of the total renewable capacity additions in 2018^22^. Figure 4 shows the resulting global rooftop solar potential map, which reports the yearly solar potential per pixel in the range of 0–10 GWh/year at a spatial resolution of 250 m  × 250 m. Two selected areas, Cairo and Delhi, are zoomed in for a better view. Based on such a global map of this type, we can plan and place rooftop solar infrastructures to their best advantage at any place on Earth. Of course, this analysis could only serve as a proof of concept from a macro perspective, for detailed analysis, it is recommended to take into account the orientation of the roof segments and roof superstructures^26^.

### Perspectives

A global map of building footprints can facilitate ongoing efforts to understand human activities, the global urbanization process, socioeconomic phenomena, and the anthropogenic impacts and human risks of climate change. As an example, almost every population density estimation study from national to global scales relies on the data published by the United Nations^27^, the European Union’s JRC^28^, or Oak Ridge National Laboratory’s LandScan^29^. However, even the United Nations and the national censuses admit that current surveying methods have intrinsic issues, calling the reliability of these studies into question. To date, applied population surveying methods do not incorporate the spatial detail of individual buildings. The best approximation methods so far are random sampling methods and extrapolation models. While current work only qualitatively describes the challenges or shows deviations from official numbers in local sample areas, new building footprints can overcome many shortcomings of these methods. Incorporating such a map of individual buildings can reduce the uncertainty of current population estimates on a global scale.

A further example is analysis of vulnerability to natural hazards and extreme events. Natural hazards, such as floods, earthquakes, landslides, hurricanes, and tsunamis, can cause catastrophic damage and significant socioeconomic loss. For example, record-shattering rainfall caused deadly flooding across Germany and Belgium in July 2021^30^. Severe floods of similar scale also now frequently take place worldwide, such as in Zhengzhou, China in July 2021^31^, and in eastern Australia in March 2022^32^. Under continued global warming, extreme events will continue to rise in frequency, intensity, duration, and spatial extent over the next decades^33^. For example, Thiery *et al*.^34^ estimate that children born in 2020 will experience a two- to sevenfold increase in extreme events, particularly heat waves, compared with people born in 1960, under current climate policy pledges. To accelerate the response to those events, especially for areas with severe damage, it is thus very important to identify vulnerable building areas well in advance. This can be realized by combining geospatial vulnerability data, digital elevation models, and building footprints. Via our example of solar potential analysis, we wish to stimulate wide usage of such unprecedented geoinformation.

Limited to the spatial resolution of global yet affordable satellite data, the accuracy of GBM has room for further improvement, e.g., by employing image super-resolution techniques prior to the segmentation of buildings, especially in places like Africa. In addition, building instances could be segmented along with the building map, which would allow for the polygonization of the footprints of individual buildings. Furthermore, high-resolution building maps updated every five to ten years would be highly relevant for many applications, which would require engagement of a larger science community. Our code and data would offer a good starting point.

Our study demonstrates the high potential and benefits of combining big data acquired by Earth observation (EO) satellites and advanced analytical methods such as deep learning. Going beyond a single use case, this is true for many geographical applications. We live in a golden era of EO with hundreds of petabytes of EO data openly and freely accessible to everyone. This big EO data offers valuable new capabilities in monitoring the changing planet, making predictions with unprecedented spatial and temporal resolution, and providing unique insights into sub-grid-scale processes in the Earth system models that have to be parameterized^35^. This opportunity, however, comes with serious challenges, related to data analytics, computational cost, and data volumes^36^, to name a few. Further community efforts are needed to develop tailored machine learning/deep learning methods and big data analytic pipelines that consider domain-specific challenges, such as domain shifts across sensors or geographical regions, sparse, imbalanced, and erroneous labels, multisensory data, generalizability and transferability issues, uncertainty quantification, physics-aware machine learning, and computationally-efficient inference^37^. Advances in these key areas will support the full exploitation of the EO data revolution going forward.

## Methods

**Fig. 5:**
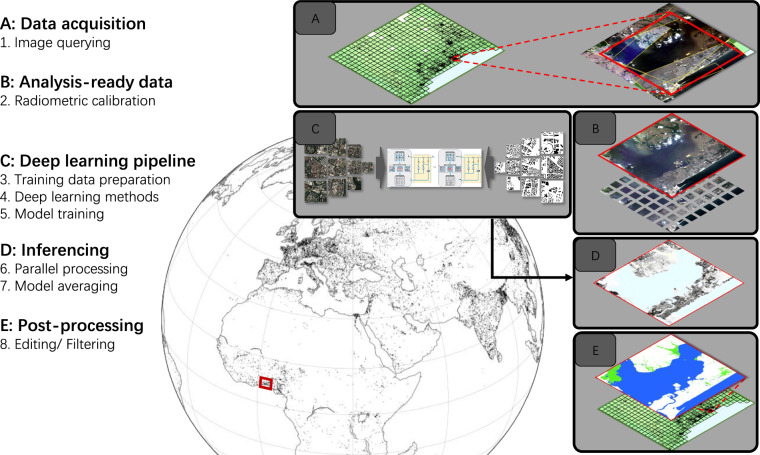
The big data analytics approach used to generate building footprints at a global scale. The workflow can be briefly summarized into five steps: data acquisition, analysis-ready data preparation, deep learning pipeline, inferencing, and post-processing. We used the Global Urban Footprint (GUF) to detect built-up areas on a 0.2° × 0.2° grid. Planet APIs were employed to acquire either Surface Reflectance or Basemap images based on the cloud coverage. Those products were calibrated and mosaicked for each 0.2° × 0.2° urban cell. The inferencing step took those mosaics and four pretrained models as input, and produced the raw global building maps. Post-processing included filtering false positives in non-urban areas with land cover layers and visualization. Image patches © Planet Labs.

This study established a framework for the mapping of global buildings from high-resolution satellite images, as illustrated in Fig. 5. This was only possible recently, owing to advances in big data analytics and the availability of high-resolution satellite imagery at relatively low cost. Four convolutional neural networks (CNNs) were utilized to detect buildings in PlanetScope satellite images at 3 m spatial resolution. We collected image patches and publicly-available OpenStreetMap (OSM)^12^ building masks from 74 cities across different continents as training data. Due to image cloud coverage and OSM incompleteness or building construction, all patches were manually inspected. Finally, 116,312 quality pairs of satellite image patch and reference building mask were used for the supervised training of four CNN models, which were subsequently used to predict buildings across the entire world. The final GlobalBuildingMap (GBM) was derived from the majority voting of the results predicted by four different models. Additionally, we utilized the resulting GBM for further analysis and derived several raster maps from it. We estimate the global PV power potential that can be derived from global buildings and solar irradiance. We also calculated building density globally.

### Data Acquisition

We collected nearly 800,000 satellite images from the Planet satellite constellation, which delivers high-resolution (3 m/pixel) optical imagery (red, green, blue, and near-infrared bands) with a high temporal revisiting frequency (up to daily).

Collection of data over global human settlements was guided by the Global Urban Footprint (GUF)^5^, a global binary map of built-up and non-built-up areas at a 12 m spatial resolution. PlanetScope images were acquired on the basis of a 0.2° grid. Images were downloaded as long as a single pixel in a 0.2° grid was human settlement according to GUF. Despite the fact that some settlements might be missing in GUF, we argue that our strategy is able to guarantee a sufficient coverage because of the generous buffer size of 0.2°.

Only PlanetScope images with a cloud and haze percentage less than 10% were used in this study. The majority of queried images were acquired from 2019. When insufficient images of required quality were available in 2019, images from 2018 were downloaded instead. In the rare case where cloud- and haze-free imagery was unavailable for both 2019 and 2018, a lower resolution basemap was used instead. The volume and number of scenes of the acquired data are given in Table 2.

**Table 2:** Volume and number of PlanetScope satellite scenes for different continents.

Region	Volume [TB]	# scenes
Africa	42	211,750
Europe	20	81,995
North America	28	119,775
South America	24	106,945
Oceania	12	61,602
East Asia	30	57,413
West Asia	13	139,592
World	169	779,072

### Analysis-Ready Data

The spectral values in each band of PlanetScope imagery come in a wide dynamic range among different cities. Extreme values of pixel intensity and highly compressed image histograms often occur. This is caused by a variety of reasons including different illumination conditions and different building construction materials. Considering that unscaled input variables can result in a slow or unstable learning process^38^, we implemented a radiometric calibration method based on robustly scaling and clipping the histogram of each spectral band. In order to retain as much information about the histogram as possible, we robustly estimated the scale and the main body of the distribution using the interquartile range (IQR). IQR is the difference between the 75th (*Q*_3_) and 25th (*Q*_1_) quartiles of the distribution, and is a commonly used robust measure of scale. For example, *Q*_1_ − 1.5 ⋅ IQR and *Q*_3_ + 1.5 ⋅ IQR correspond to approximately *μ* ± 3*σ* in a normal distribution. For heavily-tailed distributions like some bands in the Planet imagery, we were able to keep the bulk of the pixel values and then clip off the extreme values. Since the distribution of the pixel value is primarily heavily tailed, the clipping range was set from 0 to *Q*_3_ + 1.5 ⋅ IQR in our radiometric calibration, meaning only extremely large values were clipped. Afterward, all pixel values were normalized to the range 0–1. By clipping extreme values using IQR instead of a fixed percentile, we were able to adaptively control the amount of information being discarded. Our experiments show its robustness for data preprocessing at a large scale where satellite imagery covers the majority of the globe. Finally, we mosaicked all images and cropped them to individual tiles with a size of 5° in latitude and longitude.

### Deep Learning Pipeline

**Fig. 6:**
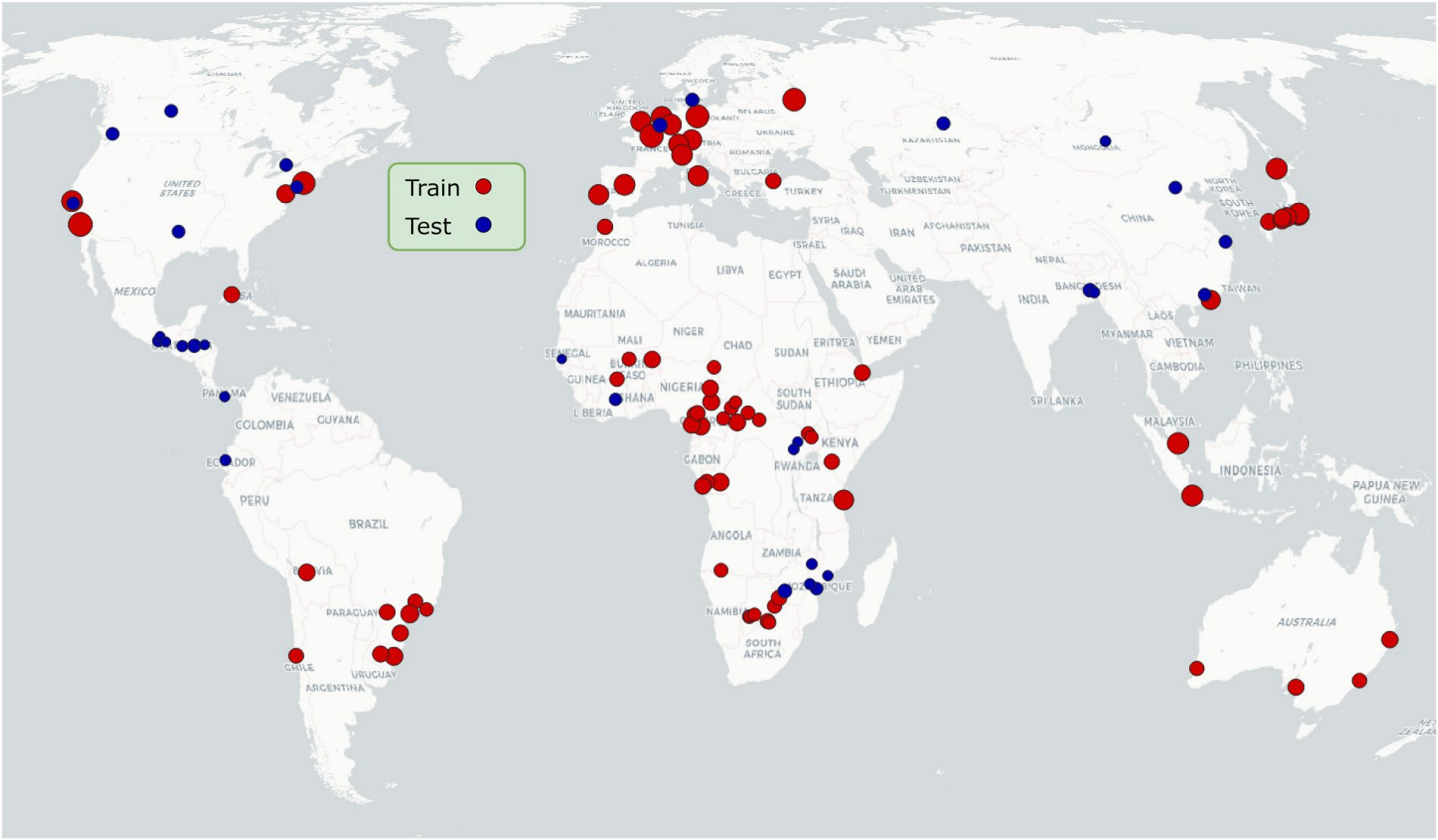
The 74 training and 34 testing cities used in this work. The data are distributed over all the continents except Antarctica. Dot size is proportional to the number of sampled patches. Background map © CartoDB.

For training data preparation, we selected Planet satellite imagery and the corresponding building footprints (stored as polygon shapefiles) from OpenStreetMap, where the detailed building footprints around 74 cities (see Fig. 6) are publicly released. Vector-format building masks were then rasterized to the same resolution as the corresponding satellite imagery for each city. Together with PlanetScope imagery, the rasterized building footprints were cropped to patches of 256 × 256 pixels.

We observed two major issues of using OSM building footprints and PlanetScope images as training pairs. The first was the mismatch of the two in some areas due to the incorrectness and incompleteness of OSM building footprints. For example, a building might appear in the satellite image, while it was missing in the corresponding OSM building footprints, or vice versa. A team of student research assistants was hired to assist with this data cleaning process, and all image/label pairs were inspected by at least two people to ensure consistency. All erroneous pairs that did not meet our strict criteria for building existence and geometric fidelity were discarded via manual inspection. The second issue was the misalignment between certain pairs, as building footprints in OSM are derived from various data sources that are different in geolocation accuracy. Therefore, coregistration was implemented in order to align the satellite imagery with the corresponding building footprints in OSM. To find the translation, satellite imagery was first transformed into a grayscale image, and a Sobel operator^39^ was then applied to both the grayscale satellite image and its corresponding building footprints. Finally, a cross-correlation was calculated between the two, and the maximum of the cross-correlation indicated the offsets between these two data sources, which correspond to the translation coefficients. The dataset was then separated into two parts, where 80% of the sample patches were used for training the network and 20% (23,287) were used for model validation.

Instead of the typical binary class label (1 as “building” and 0 as “non-building”), a truncated signed distance label was utilized as the target representation for model training. By doing so, both semantic information and geometric properties of the buildings could be learned^40^. Specifically, the truncated signed distance label takes the signed distances from pixels to the boundaries of buildings into consideration, where positive values indicate building interior and negative means building exterior. Afterward, the distance is truncated at a given threshold *β*, only incorporating the pixels closest to building boundaries^40^. Here, *β* was empirically set as 10. Finally, the distance values were categorized into a number of class labels^40^. For instance, 11 classes with the labels *L* = {0, 1, 2, . . . , 10} were categorized in our research. When the class label is larger than 5, this pixel belongs to the building, and vice-versa. The truncated signed distance label is able to capture both semantic information and implicit geometric properties of each pixel.

In our research, the task of building footprint generation belongs to the branch of semantic segmentation in computer vision, which assigns each pixel with a label (building or non-building). Convolutional Neural Networks (CNNs) effectively solve semantic segmentation problems, as they are capable of learning an enhanced feature representation directly from raw input. CNNs can get rid of heuristic feature design procedures and achieve better generalization capabilities than other traditional methods. Encoder-decoder-based networks are commonly used architectures, where satellite imagery is mapped into highly efficient feature representations in the encoder and then recovered into a segmentation map in the decoder. In our research, three encoder-decoder-based networks were selected: Graph CNN^17^, FC-DenseNet^18^, and Eff-UNet^19^, which have proven to be successful for the task of building footprint generation. Graph CNN integrates the Graph Convolutional Network (GCN) and Deep Structured Feature Embedding (DSFE) into an end-to-end workflow, where DFSE is utilized to extract more representative features and GCN aggregates the information from neighbor pixels to learn about local structures. More specifically, FC-DenseNet as a feature extractor in DSFE provides comprehensive features for a gated GCN that is proposed to model both local and global contextual dependencies. This helps to preserve sharp building boundaries and fine-grained pixel-level predictions. Both the encoder and decoder in FC-DenseNet are composed of five dense blocks, and each dense block has five convolutional layers. The key element of FC-DenseNet is the DenseNet^41^ block, which combines features using iterative concatenation. By doing so, a more efficient flow of information can be provided through network learning to improve semantic segmentation results. The encoder of Eff-UNet is EfficientNet^42^, which can efficiently learn feature maps. The decoder of Eff-UNet is comprised of five transposed convolutional layers that upsample the convolved image to predict segmentation masks. The advantages of Eff-UNet can be attributed to its capability of systematically improving performance with all compound coefficients of the architecture (width, depth, and image resolution) balanced. Moreover, it consumes much less training and inference time than the other two competitors.

The above-mentioned three networks were trained using *per city*-normalized training data. The FC-DenseNet network was additionally trained using *per patch*-normalized training data (i.e., the radiometric calibration was applied on individual patches). This approach may be beneficial to the inference stage where the spectral range of individual cities is unknown. In total, four CNN models were trained. All models were trained for 150 epochs, using a stochastic gradient descent (SGD) optimizer with a learning rate of 10^−5^. The training batch size of all models was set as 4. Negative log-likelihood loss was used as the loss function for all models.

### Inferencing

Since the volume of data to be processed was extremely large, a distributed data-parallelization (DDP) scheme was applied during inference time. We first computed the cross product between four different models and all input images. Each combination was then used to form process pools, and each process was assigned to a GPU in round-robin fashion. In order to further speed up the processing, the batch size during inferencing was maximized according to the specification of the GPU accelerator. Meanwhile, the number of workers was optimized by PyTorch. Eight NVIDIA GPUs were employed for inferencing.

Instead of relying on a single model to make predictions, we used an ensemble of all four models. Final determination of predicted class was made by the majority vote between all models. A pixel was classified as building if and only if two or more models predicted it as such. By doing so, we mitigated the randomness of model training, as well as enhanced the generalization capability of the models. This was especially beneficial for areas with high prediction uncertainty.

### Post-Processing

In order to reduce the misclassified building pixels, two land cover layers were applied to the predicted global building maps. One is the WSF layer, which is the binary land cover mask of urban and non-urban. The WSF layer is used to coarsely distinguish the urban and non-urban areas. Further, another layer, namely the 10 m “finer resolution observation and monitoring-global land cover” (FROM-GLC10)^21^ is applied for filtering, which contains nine classes, including cropland, forest, grass, shrub, water, impervious, bare land, snow, and cloud. Since false positives mainly exists in non-urban areas, we introduce area-aware filtering criteria. For urban areas, a weak filter is applied, which only filters out classes like cropland, grass, and shrub. For non-urban areas, all classes are used for filtering except for the impervious class.

## Data Records

GlobalBuildingMap^43^ is distributed on mediaTUM as a collection of binary raster files in GeoTIFF format. Each file is 5°  ×  5° with a ground sample distance of 3 m and a WGS84 projection. Files are organized in directories by continent, with Asia being divided into ASIAEAST and ASIAWEST. File names use the following format: GBM_v1_<left>_<top>_<right>_<bottom>.tif where “left” and “right” are in degrees east or west and “top” and “bottom” are in degrees north or south. For example:

- GBM_v1_e000_n10_e005_n05.tif
- GBM_v1_e005_s05_e010_s10.tif
- GBM_v1_w010_n20_w005_n15.tif
- GBM_v1_w015_s15_w010_s20.tif

All pixel values are stored in uint8, with 0 representing “non-building” and 255 representing “building”.

Apart from the building map, the dataset also includes the global photovoltaic potential map PV_density_250_compress_nodata0.tif presented in the paper. This file is limited to 250 m resolution by the World Bank’s Solargis database resolution. All pixel values are stored in float32 with units of kWh/day.

## Technical Validation

Below, we describe the accuracy of GBM predictions using both quantitative and qualitative metrics.

### Statistical Comparison

The performance of the models and their ensemble are evaluated by two metrics, F1 score and intersection over union (IoU), over the validation set in 74 cities. Although FC-DenseNet with per-patch normalization has the weakest performance on the validation set, it actually has the strongest performance on the test set. We theorize that this is due to large brightness variations within cities. The train and validation sets are sourced from the same 74 cities and divided by random split, so the model has seem images from the same cities before. However, the test cities represent a different geographic split, and are more useful in gauging model performance in unseen regions. We found the FC-DenseNet model to be superior to GCN-FSFE and Eff-UNet in our experiments.

We made further statistical comparison between our results and the building maps available from Microsoft^14^, Google^15^, WSF^8^, and GHSL^7^ on the remaining 34 test cities. The results are shown in Table 3. Although Microsoft and Google use higher resolution imagery and offer higher accuracies in many cities, neither product offers global coverage. GBM offers a higher resolution and higher accuracy than WSF and GHSL across all contients. GBM offers a notable advantage over Microsoft and Google in Europe and Asia, but falls behind in USA and Canada where Microsoft has put much of their focus.

**Table 3:** Accuracy metrics of different building map products evaluated against OpenStreetMaps data across 34 test cities distributed over five regions.

Region	# Patches	Dataset	IoU	F1	Precision	Recall	Completeness
Africa	2,708	GBM	23.3	37.8	30.1	50.7	100.0
		Microsoft	29.9	46.0	49.9	42.7	93.0
		Google	31.6	48.0	43.4	53.8	100.0
		WSF	22.1	36.3	22.3	96.0	100.0
		GHSL	17.2	29.4	19.8	56.4	100.0
Asia	3,269	GBM	38.3	55.4	45.7	70.4	100.0
		Microsoft	25.6	40.7	47.6	35.6	55.5
		Google	17.5	29.8	49.0	21.4	32.8
		WSF	32.7	49.2	33.7	91.2	100.0
		GHSL	22.8	37.2	27.7	56.3	100.0
Europe	2,605	GBM	47.9	64.8	56.0	76.7	100.0
		Microsoft	43.9	61.0	73.5	52.2	100.0
		Google	0.0	0.0	0.0	0.0	0.0
		WSF	40.6	57.7	42.5	90.2	100.0
		GHSL	26.2	41.5	36.7	47.8	100.0
Non-Latin America	3,000	GBM	46.1	63.1	51.5	81.4	100.0
		Microsoft	74.6	85.5	87.3	83.7	100.0
		Google	0.0	0.0	0.0	0.0	0.0
		WSF	34.9	51.7	35.6	94.2	100.0
		GHSL	25.0	39.9	28.8	65.2	100.0
Latin America	1,536	GBM	36.1	53.0	43.5	68.0	100.0
		Microsoft	6.8	12.8	57.2	7.2	11.6
		Google	41.9	59.1	52.9	67.0	100.0
		WSF	31.8	48.2	32.9	90.2	100.0
		GHSL	25.4	40.6	30.5	60.5	100.0
Global	13,118	GBM	41.0	58.2	48.5	72.7	100.0
		Microsoft	39.8	56.9	66.9	49.5	77.1
		Google	13.5	23.8	48.0	15.9	40.5
		WSF	33.8	50.5	34.8	91.8	100.0
		GHSL	23.7	38.3	29.3	55.1	100.0

Pixel-wise intersection over union (IoU), F1 score, precision, and recall are reported alongside patch-wise completeness (whether or not a patch contains buildings). Note that Microsoft and Google do not offer global coverage, resulting in lower completeness. Also note that the Americas were split into Latin and Non-Latin America instead of North and South America to reflect cultural differences in building construction and due to data scarcity in South America.

### Visual Comparison

**Fig. 7:**
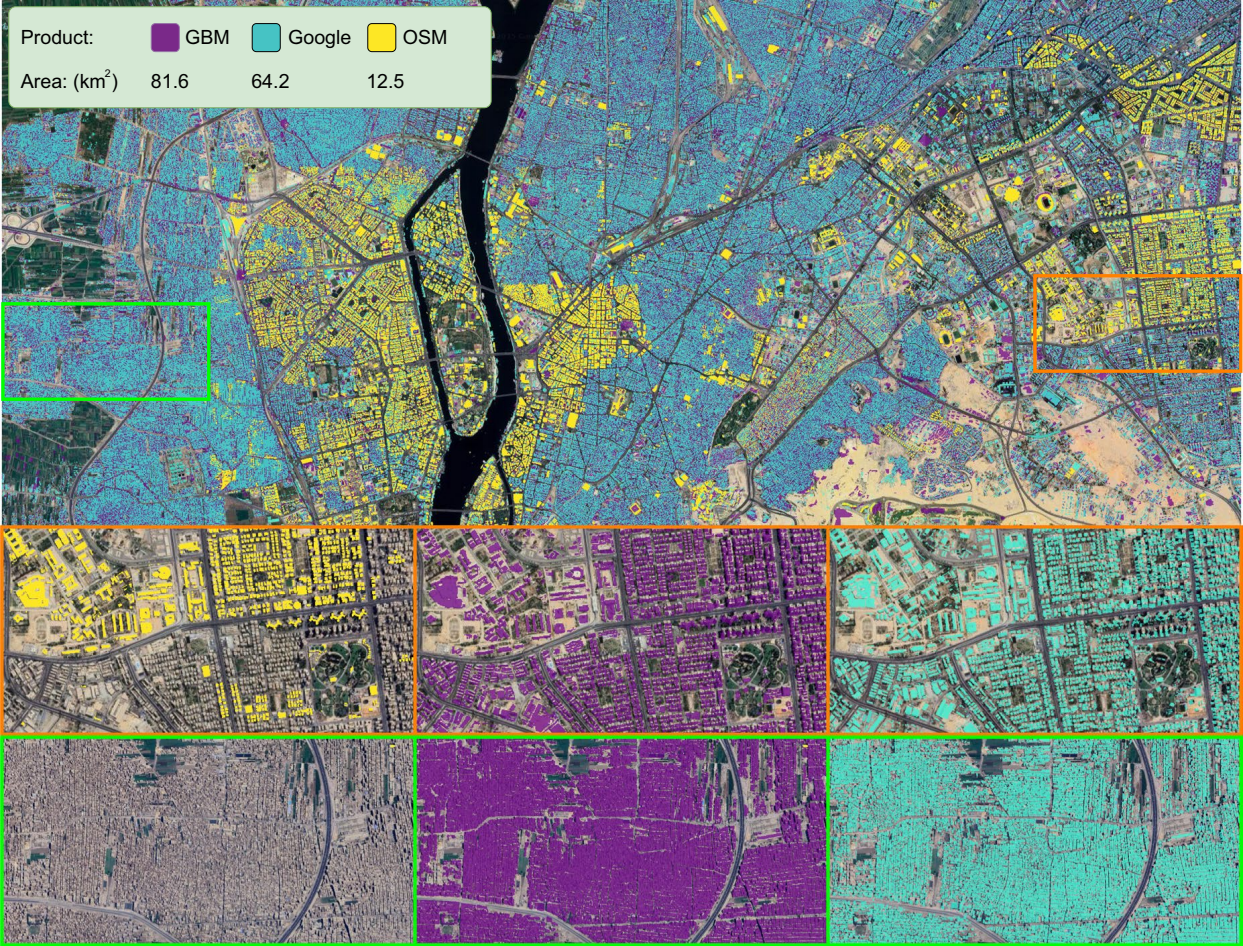
Visual comparison of building footprints from different data sources in Cairo, Egypt. The three building footprint layers from GBM (purple), Google (cyan) and OSM (yellow) are overlaid with high-resolution aerial image. Two selected areas, i.e., dense area/informal settlement (orange) and non-dense area (green) are zoomed in. Each area has three subfigures, which show the corresponding high-resolution aerial image as reference (left), GBM overlaid with satellite image (mid) and Google overlaid with satellite image (right). Background images © Google Maps.

As the most populous city in Africa, Cairo, Egypt is selected to visually exemplify building footprints from GBM, OSM, and Google in Fig. 7. In order to give a direct visual reference to where the buildings are, we use a higher resolution aerial image as the background image on which we overlay GBM (purple), Google (cyan), and OSM (yellow). Two areas, dense (orange) and non-dense (green), are zoomed in to further detail the quality of the building footprints. As expected, OSM has only limited information and does not have building footprints for most of the buildings. For a more planned area, while comparing to the higher resolution optical image (left), both GBM and Google offer very accurate building layers. Nevertheless, it can be observed that the Google building footprints miss a large number of buildings in the dense area, while GBM properly segments these buildings, despite being trained on lower resolution PlanetScope data.

### Solar Potential Calculation

The yearly solar potential was calculated using World Bank’s Solargis raster datasets^25^. These datasets are available for any location between latitudes 60°N and 50°S with a resolution of 1 km. The daily solar potential outside the latitudes was assumed with a constant value of 3.5 kWh/kWp (peak)^22^. The potential losses caused by different factors are considered according to the Solargis model. Finally, the value of every building pixel is aggregated to compute the yearly solar potential of global buildings.

## Usage Notes

GBM is distributed via the TorchGeo^44^ library. The following code can be used to construct a data loader that samples random 256 × 256 px patches from the geospatial intersection of PlanetScope images and GBM masks. The datasets will be automatically reprojected and resampled to a common coordinate reference system and ground sample distance.

from torch.utils.data import DataLoader

from torchgeo.datasets import (

GlobalBuildingMap, RasterDataset, stack_samples

)

from torchgeo.samplers import RandomGeoSampler

class PlanetScope (RasterDataset):

filename_glob = ‘*_AnalyticMS.tif’

ps = PlanetScope (paths=’...’)

gbm = GlobalBuildingMap (paths=’...’)

dataset = ps & gbm

sampler = RandomGeoSampler (dataset, size=256)

dataloader = DataLoader (

dataset, batch_size=128, sampler=sampler, collate_fn=stack_samples

)

Simply replace “...” with the directory where your data is stored, or a list of specific files.

## Supplementary information

Supplementary Information

## Data Availability

The global dataset is available on mediaTUM under a CC-BY-4.0 license: 10.14459/2024MP1764505.002. While the PlanetScope imagery required to reproduce this effort is not publicly available due to licensing restrictions, a list of all 790,101 images used in this work can be found in the https://github.com/zhu-xlab/GlobalBuildingMap/blob/main/assets/downloaded_items.txt.file. A GeoJSON file containing the bounding boxes of all processed images can also be found in https://github.com/zhu-xlab/GlobalBuildingMap/blob/main/assets/merged_roi.geojson. Note that Planet data requires a license to download.

## Code availability

All training and inference code is available on GitHub under an MIT license: https://github.com/zhu-xlab/GlobalBuildingMap.
