# Supplementary material for: GlobalBuildingMap — Unveiling the mystery of global buildings
Source: Sci Data. 2026 Jan 16;13:71. doi: 10.1038/s41597-026-06578-9 (PMC12820115; doi:10.1038/s41597-026-06578-9)
Supplement: Supplementary file 1 — Supplementary Information [file 41597_2026_6578_MOESM1_ESM.pdf]

GlobalBuildingMap — Unveiling the mystery of global buildings: supplementary information

Xiao Xiang Zhu[1,2*], Qingyu Li[1], Yilei Shi[3], Yuanyuan Wang[1], Adam J. Stewart[1,2], Jonathan Prexl[1], Fahong Zhang[1]

[1]Chair of Data Science in Earth Observation, Technical University of Munich
[2]Munich Center for Machine Learning
[3]School of Engineering and Design, Technical University of Munich
[*]Corresponding author. E-mail: xiaoxiang.zhu@tum.de

Definitions

One of the issues with cross-comparison of different building maps is the lack of clarity on what constitutes a "building". Most of the related works either have conflicting definitions of buildings or no definitions at all. These conflicting definitions often pertain to whether or not temporary shelters and slums are included, and are of vital importance to a number of potential applications. Below, we describe what information we could find about the definitions used in each product.

GUF

Global Urban Footprint (GUF) is a map of *built-up area*, which they define as "a region featuring man-made building structures with a vertical component".

HRSL

High-Resolution Settlement Layer (HRSL) is a map of *settled* regions, which they define as "containing buildings".

GHSL

Among the related works, Global Human Settlement Layer (GHSL) has the most clear definitions used in their products. They use the following definitions:

building "any roofed structure erected above ground for any use"

built-up surface / building footprint "the gross surface (including the thickness of the walls) bounded by the building wall perimeter with a spatial generalization matching the 1:10K topographic map specifications"

built-up fraction "the share of the raster sample (i.e. pixel or grid cell) surface that is covered by the built-up surface"

residential domain / residential use "the built-up surface dedicated prevalently for residential use"

non-residential domain "any built-up surface not included in the [residential domain] class"

The majority of these definitions are derived from INfrastructure for SPatial Information (INSPIRE), which makes the following definitions:

building "constructions above and/or underground which are intended or used for the shelter of humans, animals, things, the production of economic goods or the delivery of services and that refer to any structure permanently constructed or erected on its site"

residential domain / residential use "Areas used dominantly for housing of people. The forms of housing vary significantly between, and through, residential areas. These areas include single family housing, multi-family residential, or mobile homes in cities, towns and rural districts if they are not linked to primary production. It permits high density land use and low density uses. This class also includes residential areas mixed with other non-conflicting uses and other residential areas"

WSF

World Settlement Footprint (WSF) explicitly acknowledges the above-mentioned issues with unclear definitions. They use the following definitions in their WSF-2015 paper:

building: "any structure having a roof supported by columns or walls and intended for the shelter, housing, or enclosure of any individual, animal, process, equipment, goods, or materials of any kind"

building lot: "the area contained within an enclosure (e.g., wall, fence, hedge) surrounding a building or a group of buildings"

road: "any long, narrow stretch with a smoothed or paved surface, made for traveling by motor vehicle, carriage, etc., between two or more points"

paved surface: "any level horizontal surface covered with paving material"

WSF further acknowledges that different papers use different definitions of *settlement*, and provides performance metrics for three different possible definitions:

1. buildings
2. buildings + building lots
3. buildings + building lots + roads/paved surfaces

However, the authors fail to mention which of these three definitions is used in the final product.

OSM

OpenStreetMaps (OSM) explicitly excludes temporary structures, and defines a *building* as "a man-made structure with a roof, standing more or less permanently in one place".

Google

Google offers no definition of what they consider to be a building.

Microsoft

Microsoft offers no definition of what they consider to be a building.

GBM

In this paper, we follow the INSPIRE and OSM definitions of *building*, and explicitly exclude temporary shelters.
